# The *in vitro* degradation of a novel gradient-degradable ureteral stent based on copolymers of TMC and GA

**DOI:** 10.3389/fbioe.2026.1783304

**Published:** 2026-02-26

**Authors:** Ke Hu, Yu Zhang, Xinmiao Ma, Shouhua Han, Tianyang Zhou, Zhipeng Hou, Xiancheng Li

**Affiliations:** 1 Department of Urology, The Second Hospital of Dalian Medical University, Dalian, Liaoning, China; 2 Research Center for Biomedical Materials, Engineering Research Center of Ministry of Education for Minimally Invasive Gastrointestinal Endoscopic Techniques, Shengjing Hospital of China Medical University, Shenyang, China; 3 Division of Hepatobiliary and Pancreatic Surgery, Department of General Surgery, The Second Hospital of Dalian Medical University, Dalian, Liaoning, China

**Keywords:** enzymatic degradation, GA, gradient degradation, hydrolytic degradation, TMC, ureteral stent

## Abstract

Ureteral stent placement is a commonly executed clinical intervention for the treatment of upper urinary tract diseases. However, conventional non-degradable stents necessitate secondary extraction and are associated with complications such as infections, stone formation, and irritative symptoms when left indwelling for extended durations. To mitigate these risks, we introduce a novel three-layer gradient degradable ureteral stent (PTGGDG) developed from copolymers of TMC and GA [P(TMC-co-GA), abbreviated as PTG] using a multilayer impregnation technique. The structural design comprises PTG6040, PTG5050, and PTG4060 layers arranged from the inner layer to the outer layer. The present study systematically explored the relationship between the molecular chemical structure of the stent and its macroscale performance under varying proportions. The findings of this study demonstrate that PTGGDG exhibits a tensile strength of 34.69 MPa, coupled with exceptional flexibility, thus substantiating its compliance with the stringent mechanical support criteria stipulated for ureteral stents. The degradation behavior of PTG ureteral stents was rigorously assessed in artificial urine (AU) and aspergillus oryzae lipase-PBS (AP) environments. By modulating the PGA content in the formulation, the stents exhibited controllable degradation rates and maintained satisfactory morphological stability. In the present study, the stent was found to undergo predominantly hydrolytic degradation driven by GA, with the gradient structure extending functional support and minimising abrupt structural failure over a degradation period of 42 days. The PTGGDG stent undergoes preferential degradation of the outer layer in the AU due to the rapid degradation of its PTG4060 component. The middle layer’s PTG5050 plays a buffering role in this process. The inner layer’s PTG6040 degrades more slowly, ensuring a smooth lumen flow and allowing the degradation fragments to be discharged with artificial urine. This prevents obstruction of the ureteral stent fragments. In AP, the predominant form of erosion was enzymatic, driven by TMC, resulting in a degradation timeframe of 13 days. This gradient design is promising as it maintains urinary luminal patency while facilitating degradation in accordance with clinical requirements, thereby keeping urine pH within physiological ranges and providing insights for the optimization of materials and structural integrity in degradable ureteral stents.

## Graphical Abstract

**Figure:**
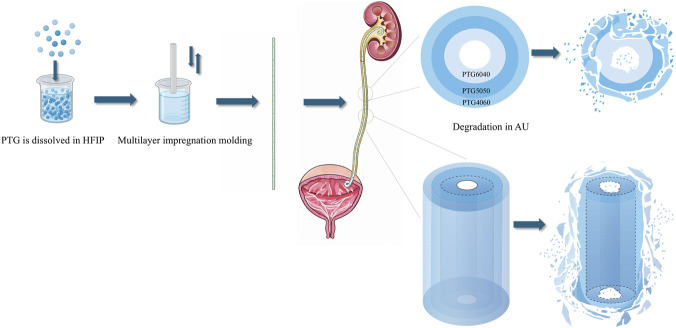

## 1 Introduction

Upper urinary tract obstruction-related diseases, including stones, strictures, tumor compression, and postoperative edema, can result in urinary blockage and heighten the risk of hydronephrosis. Consequently, ureteral stents are extensively employed to ensure adequate drainage and safeguard kidney function (Hu et al., 2024). However, conventional non-degradable ureteral stents often necessitate secondary extraction due to the potential for complications, such as the formation of stones, infections and bladder irritation. These complications underscore the necessity for research and the development of translational solutions, specifically degradable ureteral stents, which present an alternative approach to addressing these concerns.

The materials utilised in ureteral stents can be categorised broadly into two classifications: namely, non-degradable and degradable categories. The degradable category includes natural polymers, synthetic polymers, and degradable metals. In the domain of synthetic polymers, materials frequently utilised in the fabrication of ureteral stents include polylactic acid (PLA), poly(lactic-co-glycolic acid) (PLGA), and poly(L-lactide-co-ε-caprolactone) (PLCL) (Hu et al., 2024). These materials are characterized by their biocompatibility and non-immunogenic properties, ensuring that their degradation products do not provoke ureteral inflammation (Wang et al., 2018). The ideal degradable ureteral stent must be biocompatible and strong enough to preserve the tubular structure’s integrity in the body, while also degrading at a controllable rate.

Existing material systems still exhibit shortcomings; for example, PLA has limited toughness (Zhao et al., 2019). PLGA stents possess a glass transition temperature (Tg) exceeding 37 °C, which may lead to bladder irritation *in vivo*, and their high E-modulus could contribute to the risk of bladder rupture (Raya-Rivera et al., 2011). Poly(glycolic acid) (PGA) is characterized by high modulus properties, and its stiffness can be enhanced through the incorporation of typical low Tg polymers, thereby improving flexibility. Additionally, PGA is a highly hydrophilic crystalline polymer that typically undergoes rapid degradation in aqueous solutions or within the body. However, this rapid degradation can result in structural instability, the formation of acidic degradation products, and low solubility, thereby significantly restricting its application in degradable ureteral stents. In this context, the ability to achieve moderate and controllable degradation while maintaining sufficient support strength has emerged as a critical focus in material design.

Trimethylene carbonate (TMC) has attracted considerable attention on account of its exceptional biocompatibility and potential for use in the softening of brittle materials (Buchholz, 1993). The PTMC derived from TMC exhibits unique degradation behaviours, such as resistance to non-enzymatic hydrolysis, a lack of acidic byproducts during degradation, and a primary degradation mechanism characterised by surface erosion. These characteristics offer favourable prospects for the implementation of PTMC in degradable ureteral stents. Consequently, PTMC’s properties have drawn our interest. Studies have validated the use of PTMC for urethral defect reconstruction (Sartoneva et al., 2018). However, it is important to note that PTMC is prone to creep, which can result in stent displacement or dislocation (Liu et al., 2021).

In order to enhance shape stability and reduce the risk of stent displacement, it is imperative to incorporate GA, characterised by its high modulus properties, into PTMC to augment the mechanical support of the stent. This combination ensures both toughness and stiffness, while leveraging TMC’s surface erosion degradation characteristics alongside GA’s rapid degradation properties. Consequently, the stent can gradually degrade in urine environments to meet clinical requirements, while maintaining lumen patency throughout the degradation process. Moreover, TMC’s capacity to degrade without producing acidic byproducts contributes to the stability of the urinary microenvironment. Consequently, the copolymerization of GA and TMC is regarded as a viable approach for the regulation of PTG properties. There has been extensive research surrounding the synthesis and analysis of GA/TMC copolymers, which has expanded to encompass complex systems with three or more monomers (e.g., GA, TMC, CL, LA) (Joziasse et al., 2000; Kasperczyk et al., 2008; Zini et al., 2007). Notably, the widely used surgical suture Maxon is predominantly made from TMC-GA copolymer (TMC:GA = 32.5:67.5) (Katz et al., 1985), which exhibits favorable mechanical properties and a degradation period of about 7 months (Metz et al., 1990). This finding confirms the clinical applicability of such copolymerization strategies.

In addition to material composition, the manufacturing process is a pivotal factor in determining the performance of stents. Single-material or monolayer structures frequently fail to fulfil the dual requirements of providing “early stable support” and ensuring “subsequent safe degradation”. The Uriprene™ ureteral stent has faced limitations in clinical settings due to rapid fragmentation, which can lead to temporary obstruction (Hadaschik et al., 2008). In order to address this issue, a multilayer impregnation strategy is proposed for the development of a ureteral stent with a three-layer design. The outermost layer should be engineered to undergo degradation more rapidly, while the innermost layer must possess adequate mechanical properties to prevent sudden collapse during the degradation process. The configuration under discussion has been developed to prevent the formation of substantial fragments that could potentially impede the ureter and result in hydronephrosis. Prior research has demonstrated the effectiveness of multilayer impregnation techniques in ureteral stents, showing that multilayer stents made from PLGA exhibit favorable mechanical properties and biocompatibility (Yang et al., 2017).

In summary, we plan to use three different proportions of TMC and GA to copolymerize to obtain a PTG copolymer. Through the multi-layer impregnation technique, a three-layer structure, gradient-degradable ureteral stent (PTGGDG) is prepared. This stent has an “outer fast and inner slow” degradation mode. The slow degradation of the inner layer ensures the mechanical properties of the stent, preventing the sudden collapse of the stent that could cause ureteral obstruction. While the fast-degrading fragments on the outer layer can be excreted from the body through urine flushing. To date, there has been a paucity of systematic research on the development of novel gradient degradable ureteral stents derived from PTG. To the best of our knowledge, no studies have reported on the degradation behaviour of such gradient stents in artificial urine and lipase solutions. The objective of this study is to examine the *in vitro* degradation disparities of PTG copolymers at varying ratios under conditions of artificial urine and lipase. In addition, a multilayer impregnation technique will be employed to fabricate gradient degradable ureteral stents, with a view to assessing their potential applications in the field of urology.

## 2 Experimental section

### 2.1 Materials

The TMC and GA monomers were sourced from Daigang Biomaterials Co., Ltd. (purity greater than 99.5%) and can be utilized directly without additional purification. Stannous octoate (Sn(Oct)_2_), acquired from Sigma-Aldrich, was prepared as a 0.2 M solution in toluene. The lipase solution (originating from Aspergillus oryzae, ≥100,000 U/g) was also purchased from Sigma-Aldrich, stored in a refrigerator at 2 °C–4 °C, and employed as received. A hexafluoroisopropanol solution (1,1,1,3,3,3-Hexafluoro-2-propanol) was obtained from APINNO (purity greater than 99.5%) and stored at room temperature until use. All other solvents utilized were of analytical grade and processed using standard purification techniques.

### 2.2 Methods

#### 2.2.1 Preparation of PTG

The PTG copolymer was synthesized via ring-opening copolymerization of trimethylene carbonate (TMC) and glycolide (GA) using Sn(Oct)_2_ as a catalyst. The preparation process is briefly described as follows: TMC and glycolide monomers, mixed in specific molar ratios (TMC:GA = 60:40, TMC:GA = 50:50, TMC:GA = 40:60), along with the catalyst, were placed into clean, dry glass tubes. The tubes were then vacuum-sealed after being purged with nitrogen three times. Subsequently, the sealed glass tubes were immersed in an oil bath at 150 °C for 24 h. After cooling to room temperature, the glass tubes were broken open, and care was taken to remove any small glass fragments adhering to the polymer. The coarse polymer was then dissolved in hexafluoroisopropanol and precipitated in ice-cold methanol. Finally, the product was dried to a constant weight to obtain purified materials in varying ratios, designated as PTG6040, PTG5050, and PTG4060.

#### 2.2.2 ^1^H NMR

The ^1^H NMR analysis was performed on a Bruker ARX 600 spectrometer (Bruker, Zurich, Switzerland), using d6-DMSO as the solvent and tetramethylsilane (TMS) as the internal standard. The molar ratio of TMC segments was calculated based on the ^1^H NMR results. Additionally, the composition of the degradation products was also analyzed using ^1^H NMR.

#### 2.2.3 GPC

GPC measurements were conducted on an Agilent 1260 system to determine the number-average molecular weight (Mn) and molecular weight distribution (Đ_M_) of the copolymer. The system consisted of an Agilent 1260 HPLC pump, a refractive index (RI) detector, and a PLHFIPgel 300 × 7.5 mm chromatographic column. Polymethyl methacrylate (PMMA) was used as a standard, with hexafluoroisopropanol (HFIP) as the eluent at a flow rate of 1 mL/min. During the analysis, both the column and the detector were maintained at a temperature of 40 °C.

#### 2.2.4 Thermal properties

The glass transition temperature (Tg) of the polymer samples was measured using a Mettler Toledo DSC 3 equipped with a liquid nitrogen cooling system. The measurement temperature range was from −50 °C to 220 °C, with a heating rate of 10 °C/min under nitrogen flow. To eliminate the effects of thermal history, the glass transition temperature of the copolymer was measured during the second heating cycle. The thermal stability was assessed using a Netzsch TGA 209 F1 (Netzsch, Selb, Germany), where the samples were heated from 50 °C to 600 °C at a heating rate of 10 °C/min in a nitrogen atmosphere.

#### 2.2.5 FT-IR

FT-IR spectra were obtained using a Thermo Fisher Nicolet Is5 spectrometer in the wavenumber range of 4,000 to 400 cm^−1^. The measurement mode was Attenuated Total Reflectance (ATR).

#### 2.2.6 Hydrophilic and hydrophobic properties

The hydrophilic and hydrophobic properties of the copolymer were evaluated using a water contact angle goniometer (Dataphysics, Germany, model OCA 15pro). The TMC-co-GA copolymer was dissolved in hexafluoroisopropanol (HFIP) to form a thin film. At room temperature, the shape of 3 µL drops of ultrapure water placed on the sample was recorded by a camera, and the contact angle data were directly read from the computer. The measurements were conducted after waiting for 5 s following the placement of the water droplet on the polymer surface. Five different positions on each sample were measured and averaged. The microscopic morphology of the samples was obtained using an XL30ESEM-FEG microscope (FEI-Philips, Eindhoven, Netherlands).

#### 2.2.7 SEM test

The microscopic morphology of the PTD polymer material was observed using a HITACHI SU8010 microscope (Chiyoda-ku, Tokyo, Japan). Prior to testing, a gold coating was applied to the surface of the films using an ion sputtering device to enhance conductivity.

#### 2.2.8 Macroscopic morphology

The macroscopic morphology of the P(TMC-co-GA) copolymer (PTG) during the degradation process was recorded using a Huawei Mate 60 Pro.

#### 2.2.9 pH value

The pH values of artificial urine and lipase culture medium before and after degradation were monitored using a Toledo-Mettler InLabMicro TM pH meter (Toledo-Mettler, Zurich, Switzerland) equipped with a three-in-one microelectrode.

#### 2.2.10 Tensile performance

The prepared hollow ureter samples were tested on an Instron 5982 composite testing machine (Boston, Massachusetts, United States) at room temperature with a loading rate of 10 mm/min and a load range of 0–1.5 N. The samples were cut into rectangular strips with a width of 3 mm, a length of 20 mm, and a thickness of 1 mm. Each sample group was tested in parallel three times, and the average values were recorded as the results.

### 2.3 Preparation of ureteral stent

Different ratios of PTG (PTG6040, PTG5050, PTG4060) were dissolved in HFIP solution to prepare solutions at a concentration of 100 mg/mL. Quickly transfer the solution into a 5 mL silicate glass test tube and place it vertically in a test tube rack. Using an 8 cm long and 2 mm diameter polytetrafluoroethylene (PTFE) tube as the immersion mold, immerse the lower end vertically into the solution. After submerging for 5 s, withdraw it vertically at a uniform speed and expose it to standard atmospheric conditions (temperature 20 °C ± 2 °C, atmospheric pressure 101 kPa, humidity 50% ± 2%) for 1 min to allow the solvent to evaporate naturally. The PTG6040 group, PTG5050 group, and PTG4060 group repeat the above immersion process 27 times. For the gradient degradable stent group (PTGGDG) which consists of 3 layers, first immerse in PTG6040 solution 9 times, followed by immersion in PTG5050 solution for the same number of times, and finally immerse in PTG4060 solution for the same number of times. Then, expose the four groups of stent tubes to standard atmospheric pressure for 3 weeks to allow sufficient natural evaporation of the solvent. Using tweezers to hold the proximal end of the mold, wrap filter paper around the sample stent tube, and gently pull out the mold by hand.

### 2.4 *In vitro* degradation

#### 2.4.1 *In vitro* hydrolytic degradation in AU

##### 2.4.1.1 Artificial urine preparation

The artificial urine (AU) was formulated with the following concentrations: urea (12.13 g/L), sodium chloride (3.17 g/L), ammonium chloride (0.81 g/L), calcium chloride (0.35 g/L), magnesium sulfate heptahydrate (0.5 g/L), sodium bicarbonate (0.17 g/L), sodium sulfate (0.13 g/L), sodium dihydrogen phosphate (0.5 g/L), and sodium hydrogen phosphate (0.06 g/L), yielding a pH of 5.85 at 25 °C.

For the subsequent *in vitro* degradation experiments, novel ureteral stents were precisely cut into 1 cm segments from 5 cm tubes and weighed meticulously. The polymer samples were immersed in 5 mL of the AU solution, which was replenished on a weekly basis. Degradation assays were conducted in a shaking incubator at 37 °C with continuous agitation for 24 h daily. Samples were withdrawn at predetermined intervals, thoroughly rinsed with distilled water, and dried under vacuum to a constant weight. The calculation of mass loss was conducted in accordance with the stipulated formula:

$$\text{Mass loss}=\frac{\left(W_{i}{-W}_{t}\right)}{W_{i}}\times 100\%$$

$$\text{Water uptake}=\frac{\left(W_{w}{-W}_{t}\right)}{W_{i}}\times 100\%$$

W_i_ is the initial weight of the PTG copolymer material before degradation, W_t_ is the dry weight of the PTG copolymer material after degradation, and W_w_ is the wet weight of the PTG copolymer material after degradation.

#### 2.4.2 *In vitro* enzymatic degradation in AP

##### 2.4.2.1 Lipase solution preparation

The lipase solution, sourced from Aspergillus oryzae lipase, was meticulously prepared by mixing the enzyme with PBS (pH 7) in a 1:1 ratio, resulting in an AP solution.

Prior to the *in vitro* degradation analysis with artificial urine, novel ureteral stent tubes were precisely cut into 1 cm segments from 5 cm tubes and weighed with care. The polymer samples were then immersed in 1 mL of the lipase solution, with the solution refreshed daily to preserve the enzyme’s activity. Degradation experiments were conducted in a shaking incubator at 37 °C, with continuous agitation for 24 h daily. At established time points, samples were extracted from the degradation medium, rinsed with distilled water, and excess moisture was removed using filter paper. Subsequently, the samples were dried under vacuum to a constant weight, with mass loss calculated as previously described.

## 3 Results and discussion

### 3.1 Synthesis of PTG

**SCHEME 1:**
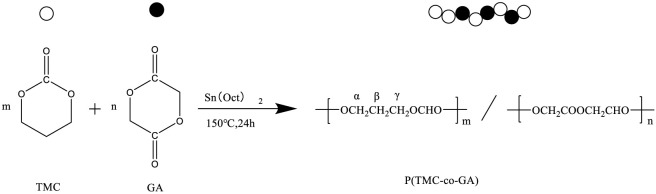
Open-loop copolymerization of TMC and GA initiated by Sn(Oct)_2_.

P(TMC-co-GA) copolymers were synthesized in differing ratios using Sn(Oct)_2_ as the catalyst in a ring-opening polymerization process (see Scheme 1; Table 1). GPC analysis showed a gradual decrease in PTG molecular weight with increased TMC content, mainly due to incomplete conversion in samples with elevated TMC levels (Zurita et al., 2006).

**TABLE 1:** Synthesis of PTG copolymers by ring-opening copolymerization of TMC and GA.

Samples	Molar ratio (TMC:GA)		M:C	Mn^b^ kDa	*Đ*_M_ ^b^	Static water contact angle	Tg^c^°C	Td^d^°C
	Feeding	Polymer^a^						
PTG6040	60:40	62:38	20000:1	70.12	1.80	47.8° ± 0.5°	18.10 °C	340.7 °C
PTG5050	50:50	50:50	20000:1	78.58	2.18	53.2° ± 0.5°	12.65 °C	338.5 °C
PTG4060	40:60	37:63	20000:1	94.92	1.90	70.2° ± 0.5°	−1.10 °C	326.7 °C

^a^ Calculated from ^1^HNMR.

^b^ Determined by GPC analysis.

^c^ Glass transition temperature.

^d^ Thermal decomposition temperature.

### 3.2 Structural characterization

**FIGURE 1:**
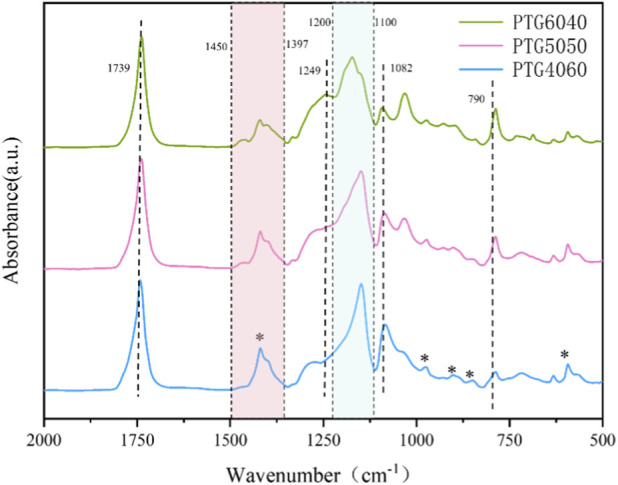
Depicts the FT-IR spectrum of PTG. Dashed boxes highlight the splitting of TMC and GA bands associated with alterations in microstructure and crystallinity. The blue dashed box illustrates the compositional evolution of the TMC band within the 1,200–1,100 cm^−1^ range, while the red dashed box signifies the compositional evolution of the GA band in the 1,450–1,397 cm^−1^ range. The black dashed lines represent the stretching vibration absorption peaks of PTG, and the asterisk (*) denotes the absorption bands linked to GA crystallinity.

The chemical structure of PTG was characterized using FT-IR and ^1^H NMR techniques. The infrared absorption spectrum in the 2,000–500 cm^−1^ range is particularly useful for analyzing the TMC and GA copolymers, as illustrated in Figure 1. The crystalline phase of PGA bands (polyglycolide bands) is marked with an asterisk (*), and the evolution of TMC (blue box) and GA (red box) bands with composition is indicated by dashed boxes (Diaz-Celorio et al., 2012). A characteristic absorption peak in the 1,450–1,397 cm^−1^ region, associated with the bending vibration (δCH_2_) of–CH_2_– in PGA segments, is most pronounced in PTG4060 and diminishes with decreasing GA content, reflecting changes in PGA proportion in the copolymer. A significant peak at 1,249 cm^−1^ corresponds to the stretching vibration of the carbonate group O–C–O in trimethyl carbonate (Hou et al., 2019), with intensity increasing with TMC content, particularly notable in PTG6040, indicating successful incorporation of TMC segments. Furthermore, characteristic peaks related to C–O–C stretching are present in the 1,200–1,100 cm^−1^ range. The peak at approximately 1,082 cm^−1^ is attributed to symmetric stretching of the ester bond C–O–C in PGA segments, with intensity rising alongside GA content; the remaining peaks also illustrate synergistic contributions of carbonate and ester bonds in the copolymer backbone. An absorption peak at approximately 790 cm^−1^, attributed to C-H bending vibrations in TMC segments, is more pronounced in samples with higher TMC content. Furthermore, the peak near 1739 cm^−1^ relates to C=O stretching vibrations in the ester, confirming the presence of ester bonds—a characteristic determined by the chemical structures of trimethyl carbonate and poly(glycolide). These findings confirm that the PTG polymer meets the anticipated molecular design specifications.

**FIGURE 2:**
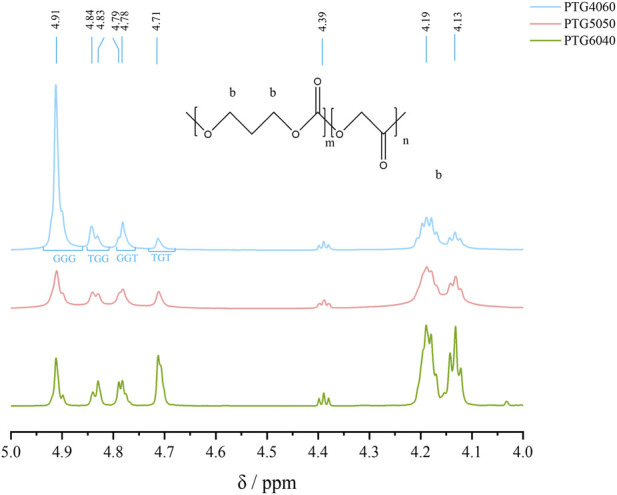
^1^H NMR spectra of PTG copolymers synthesized with different feed ratios.

^1^H NMR has been established as a powerful technique for confirming polymer structures. As illustrated in Figure 2, the chemical shift observed at 4.0–4.23 ppm is attributed to the protons of the -O-CH_2_- groups within the TMC units. The phenomenon of these peaks being separated into multiple signals indicates that the PTG copolymer is a random copolymer, rather than a block copolymer (Wang et al., 1998).

The ^1^H NMR spectra of various PTG samples demonstrate complex signals attributed to the sequence sensitivity of glycolyl protons. Four distinct triplet-associated signals, GGG (δ = 4.91 ppm), TGG (δ = 4.83–4.84 ppm), GGT (δ = 4.78–4.79 ppm), and TGT (δ = 4.71 ppm), can be clearly distinguished. The GGG triplet generates a complex signal near 4.82 ppm, characteristic of the methylene protons in the PGA polymer. The appearance of the TGT signal is primarily linked to ester exchange reactions induced by elevated temperatures and extended reaction times. Furthermore, the presence of two closely spaced signals for the TGG and TGT sequences results from tetrad sensitivity (Zurita et al., 2006).4.95–4.60 ppm for glycolyl units (G) and 4.0–4.23 ppm for the α- and γ-methylene protons (Zurita et al., 2006), as indicated at position b in Figure 2. These spectral regions were employed to calculate the actual composition of the PTG copolymer. The results reveal that the copolymer composition closely aligns with the initial feed ratio (as shown in Table 1).

As discussed, the ^1^H NMR and FT-IR analyses of the PTG copolymer are consistent with previous findings (Diaz-Celorio et al., 2012; Díaz-Celorio et al., 2013; Zang et al., 2025; Zurita et al., 2006), thereby validating the structure of the synthesized PTG random copolymer.

### 3.3 Properties of PTG

#### 3.3.1 Hydrophobicity/hydrophilicity

**FIGURE 3:**
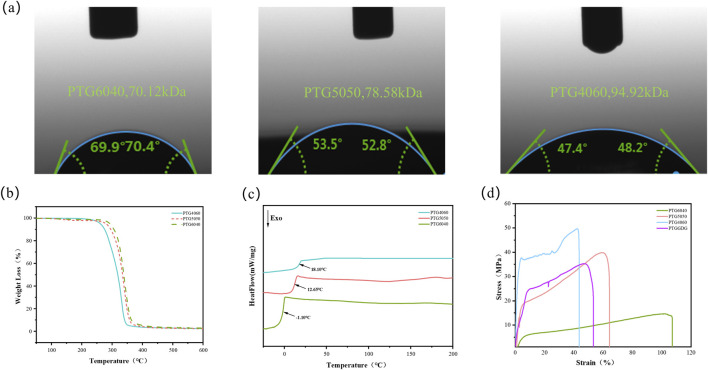
**(a)** Water contact angle graph, Scale bar = 1 mm. **(b)** TG curves. **(c)** DSC curves. **(d)** Stress - Strain curves.

The hydrophilicity of PTG copolymers was characterized using water contact angle measurements. The copolymers were initially dissolved in HFIP and subsequently cast into flat polytetrafluoroethylene molds, allowing the HFIP solvent to evaporate naturally at room temperature, resulting in PTG copolymer films. The water contact angles of these films were measured, as illustrated in Figure 3a. A notable trend was observed: as the TMC content decreased, the water contact angle of the copolymer decreased significantly from 70.2° ± 0.5°–47.8° ± 0.5°. This reduction indicates that an augmented content of GA within the copolymer matrix enhances its hydrophilicity. It has been demonstrated by preceding investigations that the incorporation of GA results in the introduction of additional hydrophilic functional groups, thereby enhancing the material’s hydrophilicity (Arbade et al., 2020; Saha and Tsuji, 2006). This enhancement is attributed chiefly to the elevated GA content, which provides a greater abundance of ester carbonyl groups. It is posited that this facilitates hydrogen bonding interactions (Schmitt et al., 1994).

#### 3.3.2 Thermal properties

The present study examined the thermal stability of PTG copolymers. As demonstrated in Figure 3b, an augmentation in the TMC segment content from 40 mol% to 60 mol% led to an escalation in the initial decomposition temperature of PTG from 326.7 °C to 340.7 °C. This substantial increase suggests that an elevated TMC content enhances the thermal stability of PTG copolymers. In addition, all PTG samples exhibited decomposition temperatures in excess of 300 °C, thus emphasising the material’s robust thermal stability. As illustrated in Figure 3c, the second DSC heating curves of PTG at varying molar ratios reveal corresponding glass transition temperatures. It is noteworthy that the glass transition temperature (Tg) of PTG exhibited a positive correlation with the increasing GA content. To be precise, the Tg increased from −1.10 °C for PTG6040 to 18.10 °C for PTG4060. The present findings are consistent with those reported in earlier studies (Diaz-Celorio et al., 2012). In our previous research, we found that PTMC homopolymers with a molecular weight of 135 kDa displayed a Tg of approximately −15 °C (Yang et al., 2015). It is evident from the comparison of the Tg of PTG copolymers that the incorporation of GA units results in a significant elevation of the Tg of PTMC homopolymers. Furthermore, all PTG copolymers exhibited a Tg value below the physiological temperature, indicating favourable flexibility for *in vivo* applications. This renders PTG suitable for the development of biodegradable ureteral stents for implantation.

#### 3.3.3 Mechanical properties

The tensile strength of polymers is primarily influenced by structural factors, such as molecular weight, as well as environmental conditions, such as temperature. The mechanical properties of PTG ureteral stents with varying ratios are illustrated in Figure 3d. The results indicate that as the GA content increases, the tensile strength of PTG6040 rises from 14.49 MPa to 49.62 MPa in PTG4060, demonstrating that PTG polymers with higher GA content exhibit improved resistance to tensile stress. However, as the proportion of GA content in the sample increases, the strain of PTG6040 decreases from 106% to 44% in PTG4060, indicating that PTG polymers with a higher TMC content exhibit superior flexibility.

In order to address the intricate clinical needs within the field of urology, there is a requirement for biodegradable polymer ureteral stents that demonstrate a combination of commendable tensile strength and flexibility. The tensile strength of the stent is crucial for maintaining patency under external compressive forces, while enhanced flexibility is vital for minimising a range of clinical complications, such as lower urinary tract symptoms (LUTS), hematuria, pyuria, and ureteral fistulae. At present, the tensile strength of commercially available ureteral stents ranges from 20 to 40 MPa (Liu et al., 2021). In contrast, the gradient-degradable ureteral stent, fabricated using a multilayer impregnation approach, exhibits superior tensile strength of 34.69 MPa and remarkable flexibility of 53%. It is evident that the properties of the aforementioned materials not only satisfy the initial mechanical strength demands for ureteral stents, but also help mitigate the risks of patient discomfort.

### 3.4 *In vitro* degradation

In this investigation, we developed a gradient-degradable ureteral stent made from PTG utilizing a multilayer impregnation technique. Notably, as far as we are aware, there exists a scarcity of research pertaining to degradable ureteral stents formulated from TMC/GA copolymer systems. To clarify the impact of copolymer composition, structure, and fabrication processes on the degradation behavior of PTG across different environments, we performed *in vitro* degradation experiments in AU and AP. The degradation characteristics were comprehensively assessed through the lenses of hydrolytic and enzymatic degradation, while also investigating the potential applications and value of PTG within the field of urology.

#### 3.4.1 *In vitro* hydrolytic degradation in AU

##### 3.4.1.1 Macroscopic morphology

**FIGURE 4:**
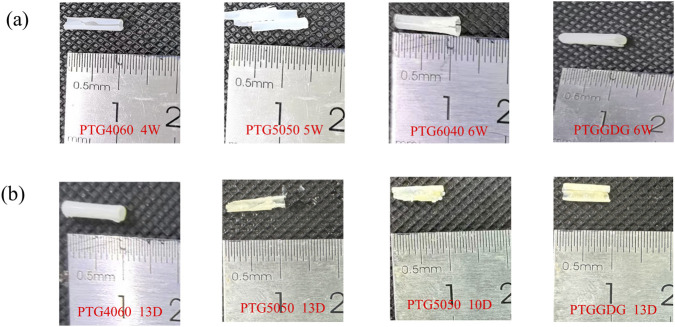
**(a)** Macroscopic morphology of the PTG copolymer demonstrating fragmentation following degradation in AU. **(b)** Macroscopic morphology of the PTG copolymer revealing fragmentation after degradation in AP.

Figure 4a illustrates the macroscopic appearance of four distinct PTG ureteral stent groups at various fragmentation time points in artificial urine. PTG4060 demonstrated complete fragmentation and a loss of supportive function by the fourth week, while PTG5050 exhibited the same by the fifth week. In contrast, PTG6040 and PTGGDG maintained their structural integrity until the sixth week. Despite the formation of cracks and the shedding of surface fragments, the PTG stents retained their overall tubular structure. This phenomenon is primarily attributable to the moderate degradation rate of the intermediate PTG5050 layer, which buffers the rapid degradation of the outer stent layer, while the slower degradation of the inner PTG6040 layer provides essential support. Consequently, by the sixth week, the macroscopic appearances of PTGGDG and PTG6040 were notably similar. Such characteristics are beneficial for ensuring ureteral patency during clinical placements, preventing sudden collapses of PGA polyester materials due to intrinsic degradation, which can lead to blockages from fragmented materials and subsequent hydronephrosis, causing iatrogenic kidney dysfunction. Uriprene™, a material developed previously, presented challenges related to unpredictable degradation rates. This resulted in the abrupt disintegration of the material and temporary obstructions in animal trials, leading to adverse outcomes (Chew et al., 2013). In response to the challenges identified, Wang et al. developed a PCL/PLGA ureteral stent. This was produced using electrospinning technology and was designed to degrade progressively from the distal bladder to the proximal kidney. The stent was developed to facilitate drainage and minimise ureteral obstruction (Wang et al., 2015). The PTGGDG ureteral stent that has been developed is uniquely designed to degrade from the exterior inward, with the PTG4060 layer degrading first. The configuration of the PTG6040 is such that it maintains its structural integrity while facilitating urine flow, thereby aiding in the clearance of surface degradation debris and potentially mitigating the formation of stone. This design may indeed signify a promising trajectory for the future development of biodegradable ureteral stents.

##### 3.4.1.2 Variations in mass loss, water absorption, pH, and molar ratio

**FIGURE 5:**
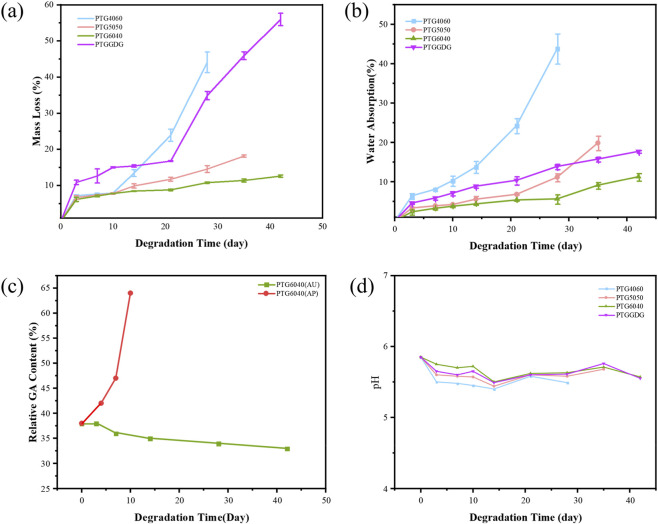
**(a)** Temporal mass loss profile of PTG samples in AU. n = 3. **(b)** Water absorption curve of PTG samples in AU. n = 3. **(c)** Temporal changes in the relative GA content of the PTG6040 copolymer in both AU and AP. **(d)** Changes in pH values of PTG samples over time in AU.

As demonstrated in Figure 5a, the PTG ureteral stent demonstrated a substantial decrease in weight during the degradation process in artificial urine. It is imperative to note that the composition of the copolymer in the ureteral stent exerts a pivotal influence on the hydrolysis rate within artificial urine. It is noteworthy that the weight change in the PTG ureteral stent demonstrated relative stability during the initial 14-day period. A significant escalation in mass loss was observed following this period, particularly evident in the PTG4060 variant. Following a 28-day period of degradation, there was a significant increase in weight loss for the PTG4060 ureteral stent, from 13.40% ± 0.95% at 10 days to 44.10% ± 2.83%. In addition, by the 28th day, the PTG4060 ureteral stent exhibited signs of fragmentation and subsequent collapse. The PTG5050 ureteral stent demonstrated signs of fragmentation on the 35th day, resulting in a mass loss of 18.11% ± 0.33%. In contrast, the PTG6040 variant demonstrated a prolonged degradation period, not fracturing until the 42nd day, with a corresponding mass loss of only 12.59% ± 0.31%, which is significantly lower than that observed in PTG4060. The degradation rate of PTG in artificial urine is primarily influenced by the proportion of GA in the stent, as the acetyl units present in GA are particularly prone to hydrolytic attack (Zurita et al., 2006). This finding suggests that increasing the GA content in the copolymer may enhance the degradation of PTG ureteral stents.

The PTGGDG ureteral stent underwent fragmentation after 42 days, resulting in a substantial weight reduction of 55.96% ± 1.70%. This phenomenon can be attributed to the composition of the stent’s outermost layer, which consists of PTG4060. Upon contact with AU, PTG4060 preferentially engages in hydrolytic degradation, which in turn exposes the innermost layer, PTG6040. Consequently, the degradation duration of the PTGGDG stent is considerably longer than that of the PTG4060 ureteral stent, resulting in a greater mass loss compared to PTG6040. An extended presence of stents in the body can elevate the risk of urinary tract infections and enhance the rate of surface encrustation (El-Hayek et al., 2017). The recommended retention time for ureteral stents is approximately 3 weeks, as this can significantly minimize the occurrence of urinary tract infections (Wang et al., 2022). Typically, the clinical implantation duration for ureteral stents is around 4–8 weeks. In contrast, the PTGGDG ureteral stent is capable of degrading within 6 weeks into small particles that can be evacuated through the urine, thereby meeting the clinical demands for ureteral stents.

Furthermore, the water absorption rates of the PTG ureteral stents were monitored throughout the degradation process, as illustrated in Figure 5b. As the degradation time increased, a rising trend in water absorption rates was exhibited by all four groups of PTG ureteral stents. By day 28, the water absorption rate of the PTG4060 ureteral stent had reached 43.79% ± 3.82%, whereas the PTG6040 group had only achieved a water absorption rate of 17.71% ± 0.31% at the time of fracture on day 42. This disparity can be attributed to the heightened hydrophilicity of PTG copolymers with higher GA content, as demonstrated in Figure 3a. The absorbed water molecules can infiltrate the material’s interior, inducing a plasticizing effect on the copolymer, which increases the mobility of the polymer chain segments and allows for enhanced hydration, promoting accelerated degradation (Edlund and Albertsson, 1999). This observation is consistent with the trend in mass loss of the PTG ureteral stents.

Figure 5c presents the relative GA content of the PTG6040 ureteral stent in AU. Over time, the relative GA content of PTG6040 exhibits a decline, which confirms that the PGA segments are preferentially degraded during the degradation process in AU.

Urinary stones are intricately linked to infections, and the pH level of urine plays a critical role in influencing the pathogens present in the urinary tract as well as the formation and progression of stones, thereby significantly affecting patient health (Khan et al., 2025). For example, an elevated pH can result in the precipitation of struvite stones, often triggered by urease produced by bacteria associated with urinary tract infections, such as those from the *Proteus* genus, or as a consequence of the presence of implanted ureteral stents (Thomas and Tolley, 2008). Conversely, a lower pH can promote uric acid precipitation in an insoluble form, which substantially raises the risk of stone formation (Kamel et al., 2002). Hence, the design of ureteral stent materials should consider the effects of their degradation products on urinary pH. Previous studies from our team have indicated that PTMC does not generate acidic degradation products during its degradation process (Yang et al., 2015). However, the hydrolysis of GA leads to the formation of acidic carboxylic products, resulting in a decrease in pH (Díaz-Celorio et al., 2013). Importantly, the PTG ureteral stent that we have developed maintains a pH range between 5.5 and 6.0 during degradation (Figure 5d), which aligns with the normal physiological range of urine pH. Although the hydrolysed degradation products of the PTG ureteral stent have been shown to reduce urine pH, they are unlikely to cause significant acidosis or tissue irritation when used in urology.

##### 3.4.1.3 SEM

**FIGURE 6:**
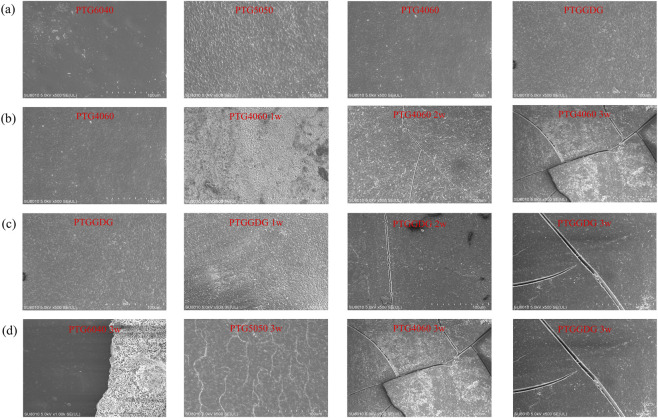
**(a)** SEM image of the ureteral stent before degradation. **(b)** Changes in the micromorphology of PTG4060 ureteral stent samples during degradation in AU over time. **(c)** Changes in the micromorphology of PTGGDG ureteral stent samples during degradation in AU over time. **(d)** Micromorphological characteristics of PTG ureteral stent samples after 3 weeks of degradation in AU. scale bar = 100 μm.

Figure 6a shows an SEM image of the stent prior to degradation. Figures 6b,c present SEM microphotographs of the surfaces of PTG4060 and PTGGDG ureteral stent samples at different degradation periods. As time progresses, the surfaces of both PTG4060 and PTGGDG samples become rougher after 1 week of hydrolysis. Cracks begin to appear in the second week, and the number of cracks significantly increases by the third week. There are more fragments in PTG4060 than in PTGGDG at the third week. This is attributed to the fact that the former degrades more quickly and experiences greater weight loss (Figure 5a). Additionally, the three-layer structure of PTGGDG provides some support from the innermost layer, PTG6040, which reduces the formation of fragments.

At the third week of degradation, we compared the surface morphologies of the four groups of samples. As illustrated in Figure 6d, the SEM microphotographs of different PTG ureteral stent samples at week three reveal their distinct surface morphologies. As the GA content in the PTG ureteral stents increases, greater corrosion is exhibited on the surfaces of the samples. As the degradation time increases, the gradual precipitation of inorganic salts from artificial urine occurs, resulting in interactions with the material surface. The PTG6040 specimen displays indications of surface encrustation, which suggests that elevated TMC content may augment the likelihood of such encrustation. This phenomenon is likely attributable to the increased presence of polar groups on the surface, thereby facilitating crystalline formation. It is noteworthy that the PTG6040 surface displays no significant corrosion, indicating that its degradation rate is lower than that of the other groups (Figure 5a). Conversely, the PTGGDG group demonstrated surface erosion by the third week, with no indications of encrustation. This reduction in encrustation risk may be attributed to the degradation of the outer layer, which has effectively diminished the potential for stone formation. This finding confirms that the multilayer structure of PTGGDG can effectively prevent encrustation while maintaining an ideal degradation rate.

##### 3.4.1.4 Changes in Mn, PDI and Tg

**FIGURE 7:**
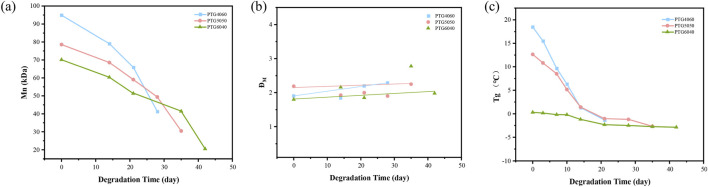
**(a)** Variation in the relative molecular weight of PTG ureteral stent samples during degradation in AU over time. **(b)** Changes in the molecular weight distribution coefficients of PTG ureteral stent samples during degradation in AU over time. **(c)** Changes in Tg of PTG ureteral stent samples during degradation in AU over time.

As the degradation time of the stent increases, the relative molecular weight of the PTG ureteral stent samples gradually decreases (Figure 7a). It is noteworthy that the relative molecular weight of the PTG ureteral stent undergoes a precipitous decline within the initial 14 days, while the associated mass loss remains comparatively negligible during this same period (see Figure 4a). This observation is in accordance with the intrinsic degradation patterns that are generally observed in polyester materials (Li et al., 2022). The findings of this study indicate that the PTG ureteral stent undergoes intrinsic degradation when immersed in artificial urine.

Further analysis of the GPC data reveals that the molar mass dispersity (Đ_M_) during hydrolytic degradation in AU shows an increasing trend (Figure 7b). This rise is due to the accumulation of low molecular weight degradation products before they dissolve (Díaz-Celorio et al., 2013).

The trend of the Tg during the degradation process of PTG ureteral stents in AU is illustrated in Figure 7c. Theoretically, as the PGA segments degrade preferentially (as depicted in Figure 5c), the Tg of the copolymer should gradually decrease over the course of degradation. As demonstrated in Figure 7c, the Tg values for PTG4060, PTG5050, and PTG6040 are consistent with the anticipated trend, suggesting a uniform behaviour among these copolymers during the degradation process.

#### 3.4.2 *In vitro* enzymatic degradation in AP

##### 3.4.2.1 Variations in mass loss, water absorption, and molar ratio

**FIGURE 8:**
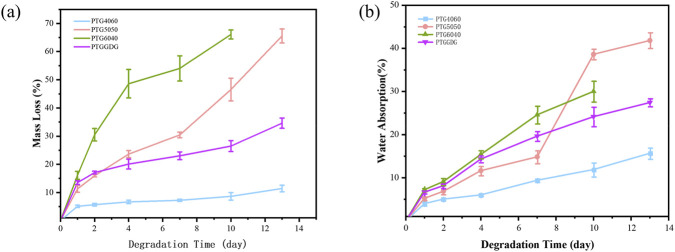
**(a)** Mass loss of PTG samples over time in AP. n = 3. **(b)** Water absorption curve of PTG samples over time in AP. n = 3.

Figure 8a illustrates the mass loss of four groups of PTG ureteral stent samples over time in AP solution. The extent of mass loss correlates with the content of the TMC segment. Notably, PTG6040 shows a mass loss of 66.12% ± 1.60% upon complete degradation by day 10, while PTG4060 only exhibits a mass loss of 8.64% ± 1.33% during the same period. Additionally, the hydrolytic mass loss recorded for PTG6040 at day 10 is relatively low, at 7.78% ± 0.20%. These results indicate that a higher concentration of TMC segments is associated with greater mass loss, and the degradation facilitated by lipase occurs at a rate faster than that of hydrolysis.

The introduction of TMC segments can significantly accelerate the enzymatic degradation rate of PTG copolymers, and the degradation rate of PTG can be modulated by increasing the TMC content in the polymer. In Table 2, referencing our previous study (Yang et al., 2015), we calculated the rate constant k for the degradation process of ureteral stents made from PTG copolymers:

$$R_{t2}=R_{t1}-k\left(t_{2}-t_{1}\right)$$

**TABLE 2:** The degradation rates of PTG samples with AP determined from the mass loss.

Samples	Rate constant k (%/w)	Correlation coefficient (R)
PTG4060	0.48	0.946
PTG5050	1.56	0.966
PTG6040	5.06	0.901
PTGGDG	4.29	0.974

In the equation, *R*_t_ represents the weight loss rate of the PTG copolymer at time t . The findings reveal that the degradation constants for PTG4060, PTG5050, PTG6040, and PTGGDG during lipase degradation are 0.48, 1.56, 5.06, and 4.29, respectively, with correlation coefficients exceeding 0.9, indicating a strong fit. Overall, the apparent enzymatic degradation rates of PTG show significant variability. Notably, as the number of GA segments increases, the degradation rate of PTG decreases. This is attributed to the fact that lipase-mediated degradation of TMC primarily occurs through surface erosion, which is influenced by the chain segments available for enzyme contact as well as the mobility of these segments. The incorporation of GA units is likely to enhance segment rigidity, thus reducing the accessibility of TMC-rich regions to enzyme active sites and consequently diminishing the number of effective cleavage sites. This observation is consistent with the findings of Liu et al. (2012). Overall, the results indicate that the degradation of PTG ureteral stents in lipase solution adheres to a controllable surface erosion mechanism that aligns with first-order kinetic equations.

Our study also recorded the water absorption characteristics of PTG ureteral stents throughout the enzymatic degradation process, as illustrated in Figure 8b. The water absorption rate of the PTG ureteral stents shows a gradual upward trend with increasing degradation time. It is noteworthy that PTG6040 achieves a water absorption rate of 30.40% ± 2.40% by day 10. In contrast, PTG4060 demonstrated the lowest water absorption rate, reaching only 15.68% ± 1.30% by day 13. Nevertheless, this rate is still higher than its hydrolytic water absorption rate of 13.80% ± 1.50% measured on day 14. This outcome suggests that lipase-mediated degradation remains the dominant process in the AP solution.

The relative GA content of the PTG6040 ureteral stent in AP is illustrated in Figure 5c. The relative GA content of PTG6040 increases with the extension of time. This confirms that TMC segments are preferentially degraded during the degradation process in AP.

##### 3.4.2.2 Macroscopic morphology

Figure 4b illustrates the macroscopic fragmentation of four groups of PTG ureteral stents at various time points in AP solution. By day 13, both PTGGDG and PTG5050 ureteral stents had completely fractured and lost their structural support. PTG6040 also completely fragmented and lost its supporting function by day 10. In contrast, PTG4060 did not show complete fragmentation by day 13. Additionally, the color of the sample surfaces transitioned from white to yellow, which may be attributed to the accumulation of degradation products from enzymatic or oxidative processes on the surface of the samples.

##### 3.4.2.3 SEM

**FIGURE 9:**
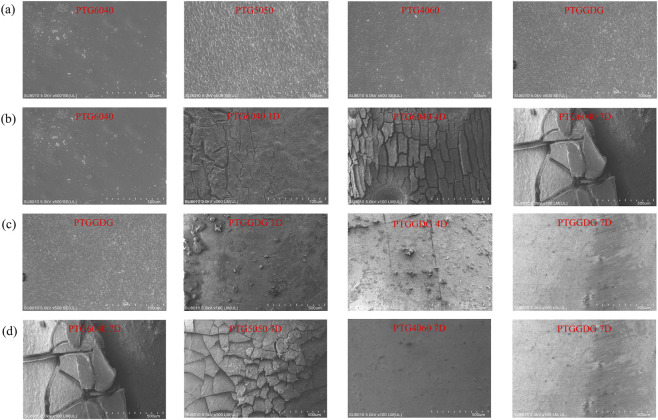
**(a)** SEM image of the ureteral stent before degradation. **(b)** Changes in the micromorphology of PTG6040 ureteral stent samples during degradation in AP over time. **(c)** Changes in the micromorphology of PTGGDG ureteral stent samples during degradation in AP over time. **(d)** Micromorphological characteristics of PTG ureteral stent samples after 3 weeks of degradation in AP. scale bar = 100 μm.

Figure 9a shows an SEM image of the stent prior to degradation. Figures 9b,c present SEM micrographs of the surfaces of PTG4060 and PTGGDG ureteral stents immersed in AP solution across various degradation periods. With the progression of time, the surfaces of both PTG6040 and PTGGDG samples became increasingly rough after just 1 day in lipase. By day 4, cracks began to appear, and by day 7, a significant increase in the number of cracks and pits was observed. Notably, PTG6040 developed more cracks and deeper pits compared to PTGGDG by day 7. This difference can be attributed to the faster degradation rate and greater mass loss of PTG6040 in the lipase environment (illustrated in Figure 8a). The observed increase in surface roughness over time further supports the presence of a surface erosion mechanism during degradation, which aligns with findings related to pure PTMC, as reported in our prior work (Yang et al., 2015).

On the 7th day of degradation, we compared the surface morphology of the four sample groups. Figure 9d presents SEM micrographs of various PTG ureteral stent samples taken at day 7, illustrating the differences in their surface morphologies. Increased TMC content in the PTG ureteral stents was associated with greater surface corrosion. The SEM findings suggest that a higher concentration of TMC segments leads to a slower degradation rate of the PTG ureteral stents, which aligns with the degradation rate constants obtained from the mass loss data.

##### 3.4.2.4 Changes in Mn, PDI and Tg

**FIGURE 10:**
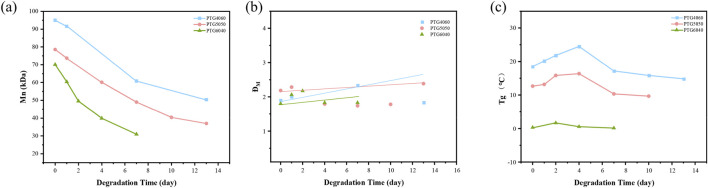
**(a)** Variation in the relative molecular weight of PTG ureteral stent samples during degradation in AP over time. **(b)** Changes in the molecular weight distribution coefficients of PTG ureteral stent samples during degradation in AP over time. **(c)** Changes in Tg of PTG ureteral stent samples during degradation in AP over time.

The changes in the relative molecular weight (Mn) and the polydispersity index (ĐM) of the samples during degradation are shown in Figures 10a,b. It can be observed that the molecular weight of the PTG ureteral stents decreases sharply within 6 days. During degradation, lipase rapidly attacks the PTMC segments, and the degradation products gradually disperse into the enzyme solution. However, when the rate of dispersion of degradation products into the AP solution is slower than the degradation process, a significant amount of degradation products will be retained in the samples, leading to a decrease in molecular weight accompanied by mass loss. For example, the amount of enzyme used is relevant since enzymes can act as surfactants to promote the diffusion of small molecular segments into the solution (Li et al., 2019; Yang et al., 2015). Although GA undergoes intrinsic degradation, the crystalline regions in PTG can hinder the diffusion and distribution of enzymes within the samples. Therefore, surface erosion degradation and incomplete intrinsic degradation coexist in PTG in the AP solution. The accumulation of small molecular degradation products on the sample surface results in a slow increase in ĐM. This is consistent with our previous findings on the enzymatic degradation mechanism of poly (2,2′-dimethyltrimethylene carbonate-co-ε-caprolactone) (Zhang et al., 2023).

Figure 10c depicts the trend of the Tg of PTG ureteral stents throughout their degradation in AP. Theoretically, as the PTMC segments preferentially degrade, the Tg of the copolymer should steadily increase during degradation, As shown in Figure 7c, the Tg values for PTG4060, PTG5050, and PTG6040 exhibit an upward trend from the beginning of degradation until day 4, at which point a subsequent decrease is observed. This initial rise in Tg is primarily due to the preferential degradation of TMC segments by lipase. Following this, the hydrolytic degradation of GA in the PBS solution occurs, leading to a decrease in Tg as the GA chain segments disintegrate. These observations reinforce the notion that PTG undergoes both surface erosion degradation and incomplete intrinsic degradation within the AP solution. Furthermore, the plasticization effect caused by the accumulation of small molecular degradation products post-degradation contributes to the later decline in Tg, consistent with findings from our earlier studies (Zang et al., 2025).

## 4 Limitations and overlook

This article is based on the PTG ureteral stent, which was prepared using the impregnation technique. Due to errors in the impregnation time, there may be minor differences in the thickness of the PTG ureters, which can lead to minor errors in the stents’ degradation cycle. Additionally, the solvent HFIP used to prepare this stent is toxic to the human body. Although the HFIP in the stent has fully volatilised, it is impossible to avoid any residual HFIP inside the stent. Therefore, biological compatibility experiments are needed to evaluate the safety of the stent. The degradation cycle of the PTGGDG stent in the body, as well as its functional gradient degradation, also need to be verified. We will continue to conduct *in vitro* and animal experiments on the PTGGDG stent in future.

The three-layer structure of the PTGGDG ureteral stent helps to reduce the risk of surface calcification and enhances overall structural stability. Its gradient layered characteristic also offers clear advantages for drug loading. In future, the middle layer could be used to load different anti-tumour, antibacterial or anti-stenosis drugs according to clinical need for the treatment of ureteral tumours, inhibition of biofilm formation on the stent surface and prevention of ureteral stenosis. At the same time, the three-layer structure can, to some extent, prevent sudden drug release, thereby achieving a more controlled release process. Research on the loading of antibacterial drugs will be carried out in the future to further verify this stent’s anti-burst release effect and anti-biofilm performance. In summary, the three-layer structure prepared by the impregnation technique and the gradient-degradable PTGGDG ureteral stent demonstrate significant potential for application and have promising prospects for clinical transformation.

## 5 Conclusion

The present study focuses on the challenges posed by clinically non-degradable ureteral stents, which often necessitate secondary removal and are at high risk of complications such as infections, stone formation, and irritation. A novel approach is proposed that employs TMC and GA to regulate the mechanical properties and degradation. Utilising Sn(Oct)2 as a catalyst for ring-opening copolymerisation, we synthesised TG6040, TG5050, and TG4060. Subsequently, a three-layer gradient stent (PTGGDG) was developed through multilayer impregnation, facilitating a degradation structure that transitions from PTG4060 on the outer layer to PTG5050 and PTG6040 on the inner layers, thus creating an “outer fast-inner slow” degradation profile. Structural characterisation confirmed that the resulting PTG is a random copolymer, a finding that is consistent with the feed ratio. Thermal analysis indicated that the Tg of all samples were below physiological temperature, which supports *in vivo* compliance. The gradient stent demonstrates a tensile strength of 34.69 MPa and a strain capacity of 53%, thus fulfilling the dual clinical demands for support and compliance. *In vitro* degradation studies revealed that in AU, hydrolysis is primarily driven by GA, with an accelerated degradation rate correlating positively with higher GA concentrations. The PTGGDG stent is designed to provide prolonged support through a progressive degradation process, thereby mitigating the risks of sudden collapse and obstruction from large particles. The overall degradation timeframe is approximately 42 days, while the pH level is maintained within the range of 5.5–6.0, which is indicative of normal human urine. Conversely, under AP conditions, the degradation is predominantly attributable to TMC-driven enzymatic surface erosion, with accelerated degradation being associated with increased TMC content. The degradation cycle in this environment is approximately 13 days. Overall, the TMC/GA gradient design presents a promising solution for achieving controllable and mild degradation while preserving lumen patency, paving the way for further optimization of materials and structures in degradable ureteral stents.

## Data Availability

The raw data supporting the conclusions of this article will be made available by the authors, without undue reservation.

## References

[B1] Arbade G. K. Srivastava J. Tripathi V. Lenka N. Patro T. U. (2020). Enhancement of hydrophilicity, biocompatibility and biodegradability of poly (ε-caprolactone) electrospun nanofiber scaffolds using poly (ethylene glycol) and poly (L-lactide-co-ε-caprolactone-co-glycolide) as additives for soft tissue engineering. J. Biomaterials Sci. Polym. Ed. 31 (13), 1648–1670. 10.1080/09205063.2020.1769799 32402230

[B2] Buchholz B. (1993). Analysis and characterization of resorbable DL-lactide-trimethylene carbonate copolyesters. J. Mater. Sci. Mater. Med. 4 (4), 381–388. 10.1007/bf00122196

[B3] Chew B. H. Paterson R. F. Clinkscales K. W. Levine B. S. Shalaby S. W. Lange D. (2013). *In vivo* evaluation of the third generation biodegradable stent: a novel approach to avoiding the forgotten stent syndrome. J. Urology 189 (2), 719–725. 10.1016/j.juro.2012.08.202 22982432

[B4] Diaz-Celorio E. Franco L. Rodriguez-Galan A. Puiggali J. (2012). Synthesis of glycolide/trimethylene carbonate copolymers: influence of microstructure on properties. Eur. Polymer Journal 48 (1), 60–73. 10.1016/j.eurpolymj.2011.10.014

[B5] Díaz-Celorio E. Franco L. Rodríguez-Galán A. Puiggalí J. (2013). Study on the hydrolytic degradation of glycolide/trimethylene carbonate copolymers having different microstructure and composition. Polym. Degradation Stability 98 (1), 133–143. 10.1016/j.polymdegradstab.2012.10.019

[B6] Edlund U. Albertsson A. C. (1999). Copolymerization and polymer blending of trimethylene carbonate and adipic anhydride for tailored drug delivery. J. Applied Polymer Science 72 (2), 227–239. 10.1002/(sici)1097-4628(19990411)72:2<227::aid-app8>3.3.co;2-q

[B7] El-Hayek G. Bangalore S. Casso Dominguez A. Devireddy C. Jaber W. Kumar G. (2017). Meta-analysis of randomized clinical trials comparing biodegradable polymer drug-eluting stent to second-generation durable polymer drug-eluting stents. JACC Cardiovasc. Interv. 10 (5), 462–473. 10.1016/j.jcin.2016.12.002 28279314

[B8] Hadaschik B. A. Paterson R. F. Fazli L. Clinkscales K. W. Shalaby S. W. Chew B. H. (2008). Investigation of a novel degradable ureteral stent in a porcine model. J. Urology 180 (3), 1161–1166. 10.1016/j.juro.2008.05.003 18639278

[B9] Hou Z. Zhang W. Guo J. Chen Z. Hu J. Yang L. (2019). The *in vitro* enzymatic degradation of poly (trimethylene carbonate-co-2, 2′-dimethyltrimethylene carbonate). Eur. Polym. J. 112, 51–59. 10.1016/j.eurpolymj.2018.12.027

[B10] Hu K. Hou Z. Huang Y. Li X. Li X. Yang L. (2024). Recent development and future application of biodegradable ureteral stents. Front. Bioeng. Biotechnol. 12, 1373130. 10.3389/fbioe.2024.1373130 38572363 PMC10987965

[B11] Joziasse C. A. Grablowitz H. Pennings A. J. (2000). Star‐shaped poly [(trimethylene carbonate)‐co‐(ε‐capro‐lactone)] and its block copolymers with lactide/glycolide: synthesis, characterization and properties. Macromol. Chem. Phys. 201 (1), 107–112. 10.1002/(sici)1521-3935(20000101)201:1<107::aid-macp107>3.0.co;2-w

[B12] Kamel K. S. Cheema-Dhadli S. Halperin M. L. (2002). Studies on the pathophysiology of the low urine pH in patients with uric acid stones. Kidney International 61 (3), 988–994. 10.1046/j.1523-1755.2002.00197.x 11849453

[B13] Kasperczyk J. Hu Y. Jaworskam J. Dobrzynski P. Wei J. Li S. (2008). Comparative study of the hydrolytic degradation of glycolide/l‐lactide/ε‐caprolactone terpolymers initiated by zirconium (IV) acetylacetonate or stannous octoate. J. Appl. Polym. Sci. 107 (5), 3258–3266. 10.1002/app.27404

[B14] Katz A. Mukherjee D. Kaganov A. Gordon S. (1985). A new synthetic monofilament absorbable suture made from polytrimethylene carbonate. Surg. Gynecol. Obstet. 161 (3), 213–222. 3898441

[B15] Khan S. A. Luo W. Wang J. Chen H. Cai Z. Qiuyu Z. (2025). Biocompatible, biodegradable, and anticancer alginate-quercetin ureteral stent via co-axial extrusion technique. Int. J. Biol. Macromol. 287, 138545. 10.1016/j.ijbiomac.2024.138545 39653206

[B16] Li X. Cui J. Liu Y. Ye F. Jin J. Xie X. (2019). The effect of molecular weight on the physical properties and *in vitro* enzymatic degradation behavior of poly (ε-caprolactone). Sci. Adv. Mater. 11 (10), 1369–1375. 10.1166/sam.2019.3419

[B17] Li K. Liu X. Di X. Bao Y. Bao Y. Xiong C. (2022). A novel biodegradable ureteral stent with antibacterial ability to inhibit biofilm formation. Mater. Adv. 3 (22), 8276–8287. 10.1039/d2ma00593j

[B18] Liu F. Yang J. Fan Z. Li S. Kasperczyk J. Dobrzynski P. (2012). Enzyme-catalyzed degradation of biodegradable polymers derived from trimethylene carbonate and glycolide by lipases from Candida Antarctica and Hog pancreas. J. Biomaterials Sci. Polym. Ed. 23 (10), 1355–1368. 10.1163/092050611X581525 21722422

[B19] Liu X. Liu S. Li K. Fan Y. Feng S. Peng L. (2021). Preparation and property evaluation of biodegradable elastomeric PTMC/PLCL networks used as ureteral stents. Colloids Surfaces A Physicochem. Eng. Aspects 630, 127550. 10.1016/j.colsurfa.2021.127550

[B20] Metz S. A. Chegini N. Masterson B. J. (1990). *In vivo* and *in vitro* degradatation of monofilament absorbable sutures, PDS® and Maxon®. Biomaterials 11 (1), 41–45. 10.1016/0142-9612(90)90050-z 2105750

[B21] Raya-Rivera A. Esquiliano D. R. Yoo J. J. Lopez-Bayghen E. Soker S. Atala A. (2011). Tissue-engineered autologous urethras for patients who need reconstruction: an observational study. Lancet 377 (9772), 1175–1182. 10.1016/S0140-6736(10)62354-9 21388673 PMC4005887

[B22] Saha S. K. Tsuji H. (2006). Hydrolytic degradation of amorphous films of l‐Lactide copolymers with glycolide and d‐Lactide. Macromol. Mater. Eng. 291 (4), 357–368. 10.1002/mame.200500386

[B23] Sartoneva R. Nordback P. H. Haimi S. Grijpma D. W. Lehto K. Rooney N. (2018). Comparison of poly (l-lactide-co-ɛ-caprolactone) and poly (trimethylene carbonate) membranes for urethral regeneration: an *in vitro* and *in vivo* study. Tissue Eng. Part A 24 (1-2), 117–127. 10.1089/ten.TEA.2016.0245 28463605

[B24] Schmitt E. A. Flanagan D. Linhardt R. J. (1994). Importance of distinct water environments in the hydrolysis of poly (DL-lactide-co-glycolide). Macromolecules 27 (3), 743–748. 10.1021/ma00081a019

[B25] Thomas B. Tolley D. (2008). Concurrent urinary tract infection and stone disease: pathogenesis, diagnosis and management. Nat. Clin. Pract. Urol. 5 (12), 668–675. 10.1038/ncpuro1254 19050709

[B26] Wang H. Dong J. H. Qiu K. Y. Gu Z. W. (1998). Synthesis of poly (1, 4‐dioxan‐2‐one‐co‐trimethylene carbonate) for application in drug delivery systems. J. Polym. Sci. Part A Polym. Chem. 36 (8), 1301–1307. 10.1002/(sici)1099-0518(199806)36:8<1301::aid-pola13>3.3.co;2-p

[B27] Wang X. Shan H. Wang J. Hou Y. Ding J. Chen Q. (2015). Characterization of nanostructured ureteral stent with gradient degradation in a porcine model. Int. J. Nanomedicine 10, 3055–3064. 10.2147/IJN.S80810 25945051 PMC4408953

[B28] Wang L. Yang G. Xie H. Chen F. (2018). Prospects for the research and application of biodegradable ureteral stents: from bench to bedside. J. Biomaterials Sci. Polym. Ed. 29 (14), 1657–1666. 10.1080/09205063.2018.1498184 30141744

[B29] Wang Y. Yang Y. Zhang H. Wang Y. (2022). Early removal of ureteral stent after kidney transplant could decrease incidence of urinary tract infection: a systematic review and meta-analysis. Exp. Clin. Transpl. 20 (1), 28–34. 10.6002/ect.2021.0183 35060446

[B30] Yang L. Li J. Zhang W. Jin Y. Zhang J. Liu Y. (2015). The degradation of poly (trimethylene carbonate) implants: the role of molecular weight and enzymes. Polym. Degrad. Stab. 122, 77–87. 10.1016/j.polymdegradstab.2015.10.016

[B31] Yang G. Xie H. Huang Y. Lv Y. Zhang M. Shang Y. (2017). Immersed multilayer biodegradable ureteral stent with reformed biodegradation: an *in vitro* experiment. J. Biomater. Appl. 31 (8), 1235–1244. 10.1177/0885328217692279 28274192

[B32] Zang T. Zhang J. Liu Y. Chen S. Han S. Guo J. (2025). Enhancing the degradation properties of poly (trimethylene carbonate) by simple and effective copolymerization of trimethylene carbonate with p-dioxanone. Polym. Degrad. Stab. 231, 111086. 10.1016/j.polymdegradstab.2024.111086

[B33] Zhang W. Hou Z. Chen S. Guo J. Hu J. Yang L. (2023). Aspergillus oryzae lipase-mediated *in vitro* enzymatic degradation of poly (2, 2′-dimethyltrimethylene carbonate-co-ε-caprolactone). Polym. Degrad. Stab. 211, 110340. 10.1016/j.polymdegradstab.2023.110340

[B34] Zhao Y. Xiong J. Shi X. Ko F. (2019). Capturing cancer cells using hyaluronic acid-immobilized electrospun random or aligned PLA nanofibers. Colloids Surfaces A Physicochem. Eng. Aspects 583, 123978. 10.1016/j.colsurfa.2019.123978

[B35] Zini E. Scandola M. Dobrzynski P. Kasperczyk J. Bero M. (2007). Shape memory behavior of novel (l-lactide− glycolide− trimethylene carbonate) terpolymers. Biomacromolecules 8 (11), 3661–3667. 10.1021/bm700773s 17941671

[B36] Zurita R. Puiggalí J. Franco L. Rodríguez‐Galán A. (2006). Copolymerization of glycolide and trimethylene carbonate. J. Polym. Sci. Part A Polym. Chem. 44 (2), 993–1013. 10.1002/pola.21199

